# Contribution of Synthetic Data Generation towards an Improved Patient Stratification in Palliative Care

**DOI:** 10.3390/jpm12081278

**Published:** 2022-08-04

**Authors:** Waldemar Hahn, Katharina Schütte, Kristian Schultz, Olaf Wolkenhauer, Martin Sedlmayr, Ulrich Schuler, Martin Eichler, Saptarshi Bej, Markus Wolfien

**Affiliations:** 1 Institute for Medical Informatics and Biometry, Faculty of Medicine Carl Gustav Carus, Technische Universität Dresden, Fetscherstraße 74, 01307 Dresden, Germany; 2 University Palliative Center, University Hospital Carl Gustav Carus, Technische Universität Dresden, Fetscherstraße 74, 01307 Dresden, Germany; 3 Department of Systems Biology and Bioinformatics, University of Rostock, Universitätsplatz 1, 18051 Rostock, Germany; 4 Leibniz-Institute for Food Systems Biology, Technical University Munich, 85354 Freising, Germany; 5 Stellenbosch Institute of Advanced Study, Wallenberg Research Centre, Stellenbosch University, Stellenbosch 7602, South Africa; 6 National Center for Tumor Diseases Dresden (NCT/UCC), Fetscherstraße 74, 01307 Dresden, Germany; 7 German Cancer Research Center (DKFZ), Im Neuenheimer Feld 280, 69120 Heidelberg, Germany; 8 Faculty of Medicine, University Hospital Carl Gustav Carus, Technische Universität Dresden, Fetscherstraße 74, 01307 Dresden, Germany; 9 Helmholtz-Zentrum Dresden-Rossendorf (HZDR), Bautzner Landstraße 400, 01328 Dresden, Germany

**Keywords:** palliative care, screening, personalized medicine, synthetic data generation, GANs

## Abstract

AI model development for synthetic data generation to improve Machine Learning (ML) methodologies is an integral part of research in Computer Science and is currently being transferred to related medical fields, such as Systems Medicine and Medical Informatics. In general, the idea of personalized decision-making support based on patient data has driven the motivation of researchers in the medical domain for more than a decade, but the overall sparsity and scarcity of data are still major limitations. This is in contrast to currently applied technology that allows us to generate and analyze patient data in diverse forms, such as tabular data on health records, medical images, genomics data, or even audio and video. One solution arising to overcome these data limitations in relation to medical records is the synthetic generation of tabular data based on real world data. Consequently, ML-assisted decision-support can be interpreted more conveniently, using more relevant patient data at hand. At a methodological level, several state-of-the-art ML algorithms generate and derive decisions from such data. However, there remain key issues that hinder a broad practical implementation in real-life clinical settings. In this review, we will give for the first time insights towards current perspectives and potential impacts of using synthetic data generation in palliative care screening because it is a challenging prime example of highly individualized, sparsely available patient information. Taken together, the reader will obtain initial starting points and suitable solutions relevant for generating and using synthetic data for ML-based screenings in palliative care and beyond.

## 1. Introduction and Definition of Palliative Care

Patients with advanced, incurable cancer suffer from changing psychological and physical symptoms in terms of type and severity. In addition, there are social burdens for both the patient and for the informal caregivers. As per the definition of the World Health Organization (WHO) (https://www.who.int/news-room/fact-sheets/detail/palliative-care (accessed on 27 July 2022)) and extended via Radbruch et al. [1], Palliative care (PC) uses a team-oriented approach to improve the quality of life of patients and their families who are facing problems associated with a life-threatening illness. It prevents and relieves suffering through the early identification, correct assessment, and treatment of pain and other problems, whether physical, psychosocial, or spiritual. Thus, it offers a support system to help patients live as actively as possible until death [1]. Furthermore, PC values patients’ needs to receive adequate, personally, and culturally sensitive information on their health status to make independent decisions about a treatment [2]. Palliative care is applicable throughout all health care settings (place of residence and institutions) and in all levels (primary to tertiary care) [3]. Primary care is performed by general practitioners, oncologists, and in outpatient structures, as well as in hospitals [4,5]. Secondary palliative care involves palliative-care specialists acting as consultants and is offered to all patients with a symptomatic advanced, progressive life-threatening disease and limited therapeutic options [6]. Furthermore, most guidelines refer to this collective [3]. Over the past five decades, PC has evolved from serving patients at the end of life into a highly specialized discipline focused on delivering supportive care to patients with life-limiting illnesses throughout the disease trajectory [4]. Still, there are different perceptions about the timing of palliative care in the course of disease, including the difficulty of a reliable and timely screening [7]. 

To the best of our knowledge, the herein presented review combining the ideas of synthetic data generation and its potential utilization towards the screening of PC needs does not exist in the current literature. Thus, we here give an introduction into both fields for an initial conjunction and motivation for the use of this quickly evolving computational field within an important medical domain, which will raise the overall awareness and open up the discussion for such novel technologies in PC or related disciplines in personalized medicine.

### 1.1. Literature Screening Methodology

We conducted our literature research in publicly available databases (PubMed, Scopus, Web of Science, Google Scholar) for the search terms of “Palliative Care” AND “Screening” AND “GANs” OR “VAE” OR “Generative Adversarial Net” OR “Variational Autoencoder” and found no matching results that could be attributed to the specific scope of this domain. As a result, we felt highly motivated to connect these two important topics.

### 1.2. Current Screening for Patients in Need for Palliative Care

Commonly, there are two screening approaches to trigger a palliative-care referral: one is based on the patient’s prognosis and the other focuses primarily on PC needs. The rationale for focusing on prognosis is that for most patients with advanced cancer symptoms, as well as others, the palliative care needs an increase within the last two months of life. The main indicators of this final phase are a poor general condition, weight loss, clinical symptoms (e.g., anorexia, breathlessness, or confusion), and abnormalities on laboratory parameters (e.g., high white cell count, lymphopenia, hyopalbuminemia, elevated lactate dehydrogenase, or C-reactive protein and Vitamin B12) [8]. The prognosis can also be derived from scores assessing physical disabilities and patient mortality based on comorbidities or the prevalence of symptoms, as well as other individual parameters [9]. A systematic review of studies using prognostic tools for identification showed that mainly five tools were evaluated for accuracy over eight studies [10]. Both sensitivity and specificity diverged widely (sensitivity 3% to 94%, specificity 26% to 99%). The authors conclude that the ability of current screening tools to identify patients with advanced progressive diseases who are likely to have palliative care needs is limited. 

The current gold standard to screen for patients’ needs is the Patient Reported Outcome Measurement (PROM) [11]. To date, several instruments are recommended for symptom assessment, e.g., MIDOS [12], ESAS [13], and IPOS [14], as well as the Distress Thermometer (DT) [15], which are described in the following. The Minimal Documentation System for Patients in Palliative Care (MIDOS) includes ten questions about distressing physical symptoms and also anxiety and depression [12]. The Edmonton Symptom Assessment System (ESAS) queries eight distressing physical symptoms and includes mood and well-being [13]. On the distress thermometer, patients can indicate their psychological distress on a scale of zero to ten [15]. The Integrated Palliative Care Outcome Scale (IPOS) represents a combination of physical symptoms with those from the psychosocial domain [14]. An integration of the outcome from such PROMs into AI-based Clinical Decision Supports Systems (CDSS) may provide a significant contribution towards the identification of PC needs, as was recently indicated by Sandham et al. [16]. To date, numerous studies have been conducted to test screening tools that combine prognostic criteria (e.g., diagnosis, functional status, complications, comorbidities) with symptoms and needs (symptom management, distress, and support of family) [17,18,19,20]. Study results show limitations in the feasibility of the tools due to time-consuming questionnaires [20]. Currently, the German SCREBEL trial compares a simple screening with a symptom assessment tool (IPOS and DT) to a more detailed assessment [17]. The era of electronic health records may facilitate referrals by providing electronic alerts, pre-populated note templates, and order sets [21]. A study performed in Würzburg currently applies the nursing history of the digital health record (nutritional status, weight loss, functional status, and unplanned hospital admissions) and combines it with PROM (ESAS, MiDOS, DT, IPOS) to assess symptoms [20]. Taken together, in routine clinical practice, screening should be reliable and require as few human resources as possible. 

To summarize, PC is an interprofessional specialty to improve quality of life for patients and their families. Existing evidence supports that timely involvement of specialist PC teams can enhance the care delivered, but identification of patients in need of PC is insufficient. International guidelines of leading medical societies recommend performing screenings as well [22,23,24,25]. However, to date, no screening tools have been developed that identify reliably those patients with individual PC needs without requiring too many medical resources. For optimal screening, heterogeneous data from different domains should be used, including both disease phase and symptoms.

### 1.3. Data-Related Challenges That Limit A General Use of AI in Palliative Care

Medical data are highly sensitive. They need proper protection and regulation. In general, data sharing is regulated under data privacy by the European General Data Protection Regulation (GDPR). With respect to the quickly evolving technology and all involved stakeholders, data sharing needs to be adequately and continuously improved by periodic adaptations of the implementations [2,26]. In terms of ethics, with the rise of novel technologies, such as Artificial Intelligence (AI), the problem also of re-identification from data, such as images and genomic information, becomes an essential aspect [27,28]. Thus, anonymization is one possibility to keep the data private. This is usually achieved by changing patient-specific identifiers through removal, substitution, distortion, generalization, or aggregation [29,30]. In contrast, data pseudonymization as another solution is a data management and de-identification procedure by which personally identifiable information fields within a data record are replaced by one or more artificial identifiers or pseudonyms [31]. Although sharing anonymized data meets the requirements of the GDPR, there have been incidents in the past where people of anonymized datasets were identified through linkage attacks [32,33]. To overcome the paucity of annotated medical data in real-world settings and (fully) save the patients’ anonymity, synthetic data generation is being used more frequently in medicine and healthcare to increase the diversity in datasets and to enhance the robustness and adaptability of AI models [34]. To conform with ethical regulations in a research context, medical data should remain only available in a highly controlled manner and according to strict procedures (e.g., ”systematic oversight” [35] or “embedded ethics” [36]). These points can be summarized as key challenges that need to be addressed:

1. *Clinical data are often few* [37,38]. The main contributing factors refer to the sparsity and scarcity of cases incident to a certain clinical problem, such as the need of palliative care that represents the very specific patient history
2. *Palliative care is a transient process and highly case specific*. There is an ongoing controversial debate on the most important parameters that are used to define and effectively screen the need for palliative care
3. *Patient data are subject to privacy issues* [2,27,28]. This hinders clinicians from sharing data with modelers, data scientists, and external clinical colleagues freely, even in an anonymized manner to improve patient classification

## 2. Existing and Prospective Applications of AI for Palliative Care

So far, research in artificial intelligence (AI) and machine learning (ML) dealing with PC have focused on survival prediction and mortality rates. To obtain an overview about these current developments, we briefly highlight and discuss the most prominent studies in the field. Random forests, feature selection, and logistic regression were applied to general patient electronic health records (EHR) [21]. In addition, a long short-term memory (LSTM) model was able to effectively predict mortality by using a combination of EHR data and administrative claims data [39]. A rapid review showed that ML approaches are powerful in predicting mortality in older and/or hospitalized adults [40]. Patients’ outcome is dependent on the right timing of specialized PC referral. Palliative patients go through different phases of their disease (stable, unstable, deteriorating, terminal/dying, deceased) [41]. Data-driven ML and network analysis were expected to identify these phases through symptoms reported on IPOS [42]. ML was moderately successful to predict cases within phases. Precision-recall curves (PRCs) were calculated in addition to ROC area under curve (AUC). PRC figures decreased from stable to terminal, leading to reduced relevance of the model for the later stages due to greater proportions of patients being in earlier palliative stages [16]. Deep learning (DL), an area of ML that uses mathematical and statistical models, has also tried to predict mortality and beneficence from PC by using a combination of clinical features including disease diagnosis and patient demographics. A Deep Neural Network model was trained on the EHR data of patients from previous years, to predict the mortality of patients within the next 3–12 month period [43]. Another study used the information on symptom burden of free-text notes in the EHR [44]. Here, natural language processing (NLP) was able to identify hospitalized cancer patients with uncontrolled symptoms (pain, dyspnea, or nausea/vomiting) in the EHR. The accuracy was between 61% and 80% with low sensitivity for nausea/vomiting (21%) and dyspnea (22%). For this reason, this model also has to be further developed before it can be used to trigger early access to PC [44]. However, despite these existing success stories, specific screening tools or CDSS of patients in need for palliative care in early, intermediate, and late stages are missing because time-specific screening parameters and a reasonable amount of underlying data are not yet available to build such tools.

A starting point for important screening features can be obtained from the National Comprehensive Cancer Network (NCCN), which has proposed consensus criteria for screening of patients care needs and subsequent referral to specialized PC: (i) uncontrolled symptoms, (ii) moderate to severe distress related to cancer diagnosis and therapy, serious comorbid physical, psychiatric, and psychosocial conditions, (iii) life expectancy of six months or less, (iv) patient or family concerns about the disease course and decision-making, and/or (v) a specific request for palliative care by the patient or family [9]. Such a systematic screening can be carried out by using checklists [45,46,47]. These included different unspecified criteria like frequent hospital admission or hospital stays due to difficult-to-control symptoms, complex nursing care, or vast deterioration. In addition, there were more specific criteria like admission from a long-term care facility or medical foster home, chronic home oxygen use, current or past hospice program enrollee, limited social support, and a lack of an advance care planning document. Others used a checklist in patients with advanced cancer stage IV, including re-hospitalization in less than 30 days, hospitalization longer than seven days, active symptoms of pain, nausea, vomiting, dyspnea, delirium, psychological distress [48]. Glare et al. [9] examined the use of six NCCN screening and further criteria (metastatic or locally advanced cancer, a limited prognosis, active source of suffering) and later included prolonged length hospital stay as an extra item [49]. Potential parameters for the screening of PC needs can thus be derived from the literature; however, the limited amount of available data across all facets is still missing.

As a supportive addition to sparse real-world data, novel synthetically generated data may serve PC in two different ways: (i) the model is trained using real-world clinical data and once trained, will not require any data in the future (fixed model approach), (ii) the model is constantly fed with data to generate synthetic data (continuous model approaches). There are three different categories of algorithms used in the generation of synthetic data: probabilistic models, machine learning, and deep learning methods. Currently, an implementation towards the field of PC screening is still missing.

## 3. Potential Impact of Synthetic Data Generation Towards an Improved Identification of Patients in Need of Palliative Care

### 3.1. Synthetic Data Generation via Generative Adversarial Networks

If only a small amount of data can be made available to the AI model, that oftentimes is not enough for optimizing, training, and testing a precise and robust decision support model at a clinical scale. Synthetic data generation would be a sensible approach to tackle this problem. Here, relevant medical data (pseudonymized, anonymized or actual) is used as an input for an ML-model to learn the underlying data structure, which is utilized in a subsequent step to generate new artificial data that is close to the original. Thus, instead of providing the AI model only with a small amount of data, a larger amount of synthetic data can be provided for the purpose to improve the training of ML-based decision support models, e.g., for patient stratification. Deep generative models, such as Variational Autoencoders (VAE) [50] and Generative Adversarial Networks (GAN) [51,52], play a key role in this. Although VAEs are also widely applied for generative modeling studies, especially with respect to sparse and scarce data in the medical/health domain for images [53,54] and data integration [55], relatively few examples for tabular data exist [56,57,58]. GANs are currently seen as most promising according to the findings of Xu et al. [57]. They see GANs as better suited for privacy preserving data generation in comparison to VAEs, since these are easier to integrate with respect to differential privacy. Several of such models have been developed over the past few years and a current technical review of Hernandez et al. [58] presents the different synthetic data generation methods for tabular healthcare datasets. A comparable work of Georges–Filteau and Cirillo investigates the possibility of synthetic data generation via GANs to ultimately obtain digital twins [59]. However, deep generative models are more popular for synthetic data generation from image datasets and there are only relatively few models relying on tabular patient data as yet [60].

**Figure 1:**
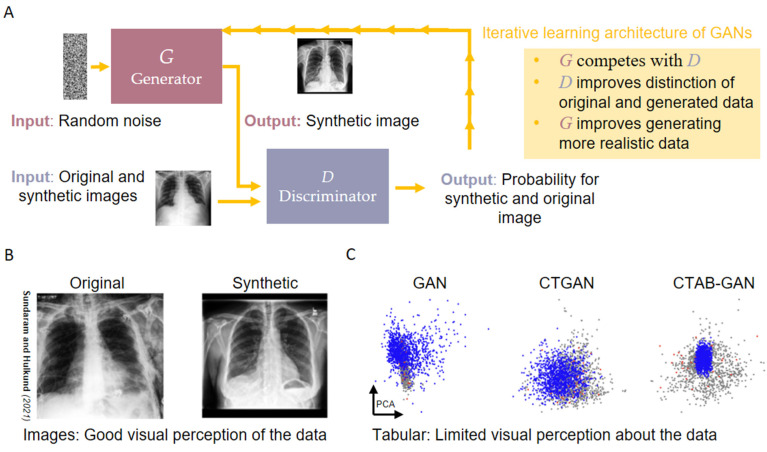
Main principle of the GAN architecture. (**A**) Refers to the iterative learning principle of Generative Adversarial Networks (GANs) using two Neural Networks (Generator G, and Discriminator D). (**B**) Shows examples of synthetically generated images, adapted from Sundaram and Hulkund [64]. (**C**) Shows a current example of a current benchmark for synthetic tabular data, adapted from Schultz et al. [65].

Traditionally, a generative network for adversarial learning consists of a Generator G and a Discriminator D (Figure 1). The Generator is realized as G: N→X, meaning that, ideally, the generative model G maps random noise to the data space, X. The Discriminator D: G(N)→[0,1] ensures that the synthetic samples generated by the Generator G are realistic enough. The two neural networks G and D compete throughout the training process with G generating synthetic samples from random noise and D ensuring that with each iteration, G learns to generate more realistic synthetic samples. However, like every neural network model, GANs require a lot of data to be trained. Thus, for smaller tabular datasets, they are often not the best option for synthetic data generation. These might be addressed by specific linear interpolation-based algorithms that take explicitly rare cases into account. Interpolation-based methods applied to small data neighborhoods are commonly used in the context of imbalance tabular datasets of smaller size. Although these methods are developed in the context of synthetic data generation to tackle class imbalance, the underlying philosophy of linear interpolation can be applied to generate synthetic data from tabular datasets, in general. Imbalanced datasets are characterized by unequal distribution of samples over classes. Since some classes have fewer examples, synthetic samples are generated for such classes to create balanced classifiers over such datasets. Our recently proposed algorithms LoRAS [61] and ProWRAS [62], as well as generalizations of the SMOTE algorithms [63], propose a way to control the variance of the synthetic samples by generating them as convex combinations of multiple shadowsamples (Gaussian noise added to original samples) from data neighborhoods. Tabular datasets typically have well-defined features following a distribution in every data neighborhood, ensuring a synthetic sample generated as a convex combination/weighted average, which is an unbiased estimator of the local mean for every feature distribution. Thus, challenges for synthetic data generation from small datasets still remain, but it is essential that these challenges are directly addressed by using real-world medical datasets to likewise identify further specific hurdles and finally ensure a versatile use on a clinical scale.

### 3.2. Domain Level Challenges Concerning the Use of GANs for Clinical Problems

For an improved, realistic representation of the current limitations, we present data and domain-related challenges in PC to motivate the importance of the conducted research in this area: Firstly, the diverse data types that are usually present in clinical tabular data, i.e., continuous and categorical data can pose a challenge in model building. In particular, categorical data highly increase the complexity because they can be further divided into nominal and ordinal data-types. This requires the ML-model to handle potentially complex continuous and discrete distributions at the same time. Additionally, continuous features can follow different distributions and have multiple modes. Secondly, considering that synthetic data generation is a feasible solution to support data privacy, the development and comparison of algorithms, metrics, and protocols that can quantify how reliably the synthetic data represent the original data would be crucial for a practical realization. Finally, the usability and technical acceptance of clinicians using the developed models are often not adequately addressed right from the beginning.

(i) Since 2017, there exist multiple deep generative models focusing on synthetic data generation on tabular datasets. MedGAN, the first of such architectures, can handle either Boolean or count data [66]. After the initial release, there were several adaptations of this architecture to enable the generation of categorical values and to boost performance (e.g., changing the loss from vanilla GAN loss to Wasserstein loss) [52,67,68]. Another model, TableGAN, proposed shortly after, is based on deep convolutional GAN (DCGAN), uses an additional third neural network called classifier that predicts labels, and can generate numerical and categorical values [67]. TGAN or tabular GAN is yet another contemporary model that handles multiple modes in continuous variables through Gaussian Mixture Models (GMM) and can create categorical values with the help of gumbel softmax as activation function [69]. It also uses a LSTM as a generator. The authors also published an improved model in 2019, called CTGAN, which is based on the conditional GAN architecture, in which conditional vectors for categorical values are introduced [57]. In comparison to TGAN, CTGANs no longer use LSTMs as the generator network. A more complete list of different GAN architectures for tabular data that were published until the end of 2020 can be found in the work of Coutinho-Almeida et al. [70]. Since then, other GAN-based architectures were proposed [71,72,73,74,75]. Among these, one interesting recent work refers to CTAB-GAN from 2021, which combines the ideas of TableGAN and CTGAN [74]. It uses convolutions and a DCGAN architecture in addition to GMM and conditional vector construction. Additionally, it adds to the sampling mechanism a random selection of the mode of multi-modal continuous variables and can handle more data types. Besides GANs, other generative approaches, like Variational Autoencoders (VAE), Classification And Regression Trees (CART), Bayesian Networks (BN), and Copulas, etc., exist. However, the flexibility of GANs to handle complex distributions, and their success in the generation of other types of data (especially images) make it one of the most promising approaches for the generation of tabular data as well.

(ii) For tabular data, there is yet no consensus in science on how to evaluate synthetic data. Therefore, it is still an open research field. Loosely, existing evaluation metrics can be divided into four categories as follows:

Firstly, the statistical similarity of generated data can be compared to real data. Since the features are consistent and well defined across all data points, statistical hypothesis tests can be used to compare feature-wise distributions among the synthetic and original data. To measure the relationships between multiple features, pairwise correlation, k-way-marginals, or results of clustering approaches can be compared. Additionally, it is also possible to compare the similarity of the joined probability distributions of all features through metrics like Wasserstein distance, Kullback–Leibler Divergence (KL divergence), or Jason–Shannon Divergence (JSD).

Secondly, the generated data can be compared to real data with a specific task in mind. Usually, the task is to predict a specific feature given all the other features (ML efficiency). Therefore, the generation model is trained on a partition of the original data and afterwards, used to generate synthetic data. A predictive model is then trained on these data. Additionally, another model is trained on the same partition of the original data, which was used to train the generative model. Both predictive models are then compared on the test set of the original data.

Thirdly, unsupervised learning approaches can also be used to assess the similarity of synthetic data in relation to the original data. In particular, tabular data contain diverse feature types, such as continuous (e.g., BMI, height—variables that can take theoretically any real value), original (e.g., patients having hypertension or diabetes—categorical variables have a sense of order associated with them), and nominal (e.g., sex of a patient—categorical variables do not have a sense of order associated with them). Recent studies indicate that a conventional application of state-of-the-art dimension reduction algorithms, like UMAP, on such heterogeneous data lead to a biased embedding generation dimension reduction, in a sense, that the similarity among data with respect to the continuous features have a higher influence in the low dimensional embedding generation. A novel empirical feature-distributed approach has been proposed by Bej et al. that accounts for this bias [76]. In brief, the method uses separate distance measures for available feature embeddings and finally, combines these into a single embedding, which is used to detect and visualize clusters in a more robust manner. This method could also be adopted to extract embeddings from the original data, which in combination with a supervised Neural Network, can be applied to assess the similarity between synthetic and original samples.

Finally, synthetic data can be evaluated regarding privacy. One possible way for this is to simulate membership inference attacks, where an attacker tries to predict which record was used for the training of the generative model. This can be done by calculating the distance to the closest record (DCR) in the real data for each synthetic record. Another way to evaluate the privacy of a given model is to perform attribute disclosure attacks, where the attacker uses a set of non-sensitive attributes to predict a sensitive attribute. To migrate the risks of privacy leakage, several techniques like differential privacy were proposed.

(iii) A successful software implementation into the clinical routine requires in addition to the expert-in-the-loop and the knowledge of the technical infrastructure (e.g., clinical information system and stored data types) also the practical aspects of usability, feasibility, and technology acceptance. This can be achieved through user-centered design (UCD) processes, which involve clinicians as the later users in the development at an early stage because this highly facilitates the acceptance and user-friendliness [77,78,79]. A UCD is described, among other things, by DIN EN ISO 9241-210, “Processes for the design of usable systems” [80]. In general, at the beginning of the UCD, the application context and the exact user requirements must be specified to be understood. These activities are carried out by means of a user-centered requirement survey (stakeholder analysis). For example, guideline-supported interviews are used for stakeholder analysis and expert workshops for the development of prototypical user interfaces.

**Figure 2:**
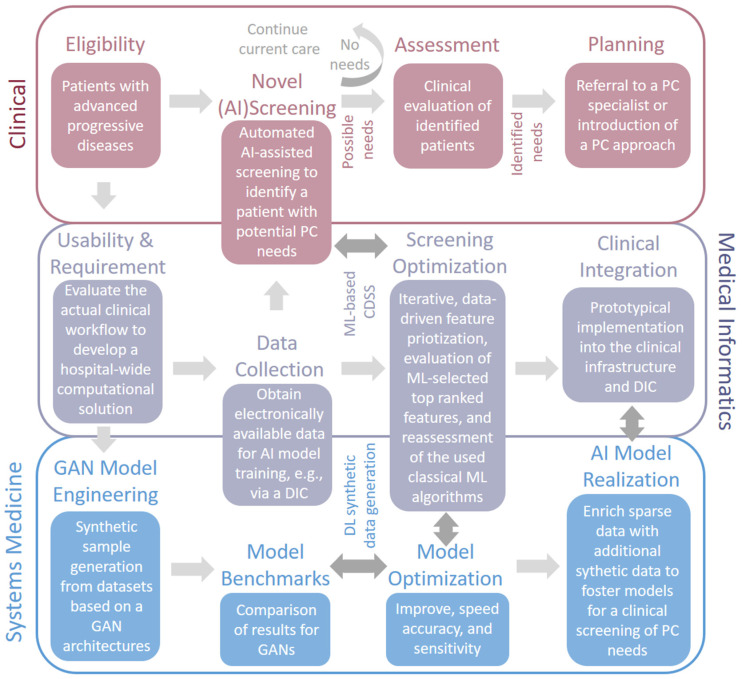
Scheme to illustrate the mutual cooperativity between the fields of Systems Medicine, Medical Informatics, and the clinical domain of Palliative Care (PC). The schematic representation is a content-wise extension of ElMokhallalati et al. [82], who only considered the clinical aspect. The specific introduction of Medical Informatics results in an advanced access of digitized medical data, e.g., through a Clinical IT Center like a Data Integration Center (DIC). Thus, underlying Artificial Intelligence (AI) approaches, i.e., Machine Learning (ML) and Deep Learning (DL), as well as Generative Adversarial Networks (GANs) for data generation, are able to foster the overall screening process in PC.

As shown above, open questions remain in the field of synthetic data generation and its application. In Figure 2, we demonstrate the potential synergistic activities and current developments in Systems Medicine and Medical Informatics to improve the clinical outcome of PC screening. The image highlights that all domains share specific, essential processes, such as Data Collection, Screening, and Optimization, because these processes need a more interdisciplinary approach. The overall aim should be an approach that can be offered to all identified patients with a symptomatic advanced, progressive life-threatening disease, and limited therapeutic options. The highest level of evidence relates to cancer patients but it would be not limited to these outcomes. Importantly, two thirds of advanced cancer patients have unmet palliative care needs [81]. Specialized algorithms for the generation of such heterogeneous tabular patient data would thus highly facilitate the early identification of actual patients leading to an actual clinical impact.

## 4. Clinical Impact of AI and GAN-Based Screening Solutions in Palliative Care

**Figure 3:**
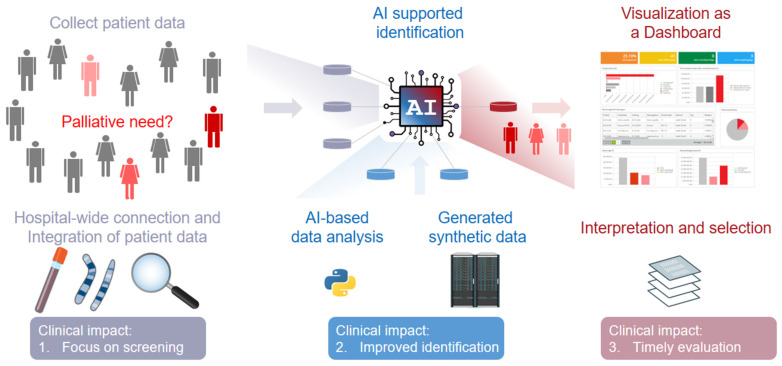
Scheme to illustrate the arising clinical impacts of synthetic data generation and Artificial Intelligence (AI) in general for palliative care. Such a concept would focus on patient screening and would use AI-based methods for an improved identification of patients, including a timelier screening (represented as different shades of red for the patients).

To assign the presented AI-based methods towards a more specific clinical outcome for palliative care screening, we summarized the computational tasks and their attribution to potentially arising clinical impacts (Figure 3). Here, the interplay and synergistic effects of the involved research areas, namely, Medical Informatics, Systems Medicine, and Clinics, for the domain can be conceived on a broader scale.

### 4.1. Clinical Impact 1: Set A Focus on Screening Rather Than Prognosis

ElMokhallalati et al. [82] conclude that existing screening tools are not adequate to represent palliative care needs, particularly because the focus is on prognosis. The rationale for focusing on prognosis is that for most patients with advanced cancer, symptoms, and thus PC, need to increase within the last two months of life [10]. Here, it would be of pivotal interest to explicitly identify those patients with an accurate screening rather than predict the remaining life expectancy or individual prognosis.

### 4.2. Clinical Impact 2: Identification of Patients with Palliative Care Needs and Its Barriers

As already pointed out, two thirds of advanced cancer patients have unmet palliative care needs [81]. A study of inpatients with and without cancer revealed that 6.9% of them had palliative care needs, but only 2% of these received specialized palliative consultation. Especially, older patients without relatives who suffered from metastatic cancer and/or liver cirrhosis had the highest risk of developing PC needs. Often, those patients only request PC themselves if they have high symptom burden. However, patients are more likely to pursue specialized PC if recommended by their oncologist [83]. Of note, oncologists can also have over optimistic estimates of survival [10], a mistaken concern about a shortening of survival [49], a misconception of PC as synonymous with end-of-life care, as well as insecurities in the communication about PC, which often results in late referrals [23,84,85,86,87]. Therefore, a physician independent screening with a recommendation to re-evaluate the individual needs can significantly support physicians to improve the treatment of patients with unmet palliative care needs.

### 4.3. Clinical Impact 3: Evaluation of the Correct Timing to Specialized Palliative Care

Early (within 2 to 3 months of diagnosis of advanced diseases) [88] provision of palliative care concomitant to life-prolonging treatment is associated with better quality of life, fewer depressive symptoms, less aggressive care at the end of life [88], and improved quality of life, symptom burden, and patient satisfaction compared to standard oncological care [22,24,25,89,90]. Contrarily, these patients are often in a good performance status [7,91,92,93,94]. A recent subgroup analysis of the early-integration Zimmermann trial showed that only patients with higher symptom burden at baseline derived a benefit from the palliative care intervention [95]. Although a too-late PC intervention may shorten survival and worsen quality of life [88], it is not possible to provide early PC for all patients with advanced disease due to the scarcity of resources [96]. Timely integration of PC is included in the European Society of Medical Oncology (ESMO) [24], as well as in the German-language palliative care guideline [97], the recommendations of the German Comprehensive Cancer Centers (CCC), and the American Society of Clinical Oncology (ASCO) recommendations for best oncology practice [7]. In summary, the importance of novel solutions is clearly given and needed.

## 5. Conclusions

Palliative care has evolved from serving patients only at the end of life into a highly specialized discipline focused on delivering supportive care to patients with life-limiting illnesses throughout their patient journey. This very individual track needs specific attention and awareness for a proper and timely screening, which is a time-intense and domain-expertise-driven process that is difficult to achieve in clinical routine at all times. Therefore, a physician-independent automatic screening, supporting the physician’s assessment, would be essential to improve the referral of patients with unmet palliative care needs. Current AI solutions already provide a well-suited tool set, but are still limited in terms of data availability and, thus, a versatile clinical applicability. A highly promising approach to filling this gap can be attributed to GAN-based synthetic data generation to provide AI classification models with an enriched set of anonymous, heterogeneous patient data to achieve likewise a high degree of data security and an accurate model performance. As was initially shown within this review article, synthetic data generation and PC have both so far only a limited number of common grounds. However, as other medical domains already show promising results and GANs are used more and more for data sharing in data sensitive domains, this review might contribute towards examples in the near future. In general, the high amount of methods and restricted consensus of evaluation metrics for synthetic data remain the main limitations that have to be solved from a computational perspective. In contrast, for PC, the main limitation is the availability of enough individual patient data, for which synthetic data could be one possible, existing solution. This novel combination can therefore lead to more precise AI-based models and finally, to improved clinical screening tools in palliative care.
